# Can virtual-reality simulation ensure transthoracic echocardiography skills before trainees examine patients?

**DOI:** 10.5116/ijme.6321.8e5d

**Published:** 2022-09-30

**Authors:** Martine S. Nielsen, Jesper H. Clausen, Joachim Hoffmann-Petersen, Lars Konge, Anders B. Nielsen

**Affiliations:** 1 SimC - Simulation Center, Odense University Hospital, Odense, Denmark; 2 Department of Anesthesiology and Intensive Care, Odense University Hospital, Svendborg, Denmark; 3 Copenhagen Academy for Medical Education and Simulation, The Capital Region of Denmark, Copenhagen, Denmark

**Keywords:** TTE, transthoracic echocardiography, assessment tool, simulation-based training, medical education

## Abstract

**Objectives:**

This study aimed to develop and gather the validity evidence for a standardised simulation-based skills test in transthoracic echocardiography and to establish a credible pass/fail score.

**Methods:**

Experts developed a virtual-reality simulator test in cardiology, medical education and simulation-based education. Thirty-six physicians with different experiences in transthoracic echocardiography completed the test at Odense University Hospital, Denmark. The performances of novice, intermediate and experienced participants were compared using the Bonferroni post hoc test. Cronbach's alpha was used to determine the internal consistency reliability of the test. The consistency of performance was analysed using the intraclass correlation coefficient. A pass/fail score was established using the contrasting groups' standard-setting method.

**Results:**

We developed a test with high consistent reliability (Alpha = .81), 95% CI [.69, .89]. In both cases, the performers’ level was consistent, fitting others at the same level of experience (intraclass correlation r_(35)_=.81, p<.001). A pass/fail score of 48/50 points was established based on the mean test score of novice and experienced physicians.

**Conclusions:**

We developed a standardised virtual-reality simulation-based test of echocardiography skills with the ability to distinguish between participants with different levels of transthoracic echocardiography experience. This test could direct a mastery learning training program where trainees practise until they reach the pre-defined level and secure a higher level of competency to ensure quality and safety for patients.

## Introduction

Transthoracic echocardiography (TTE) is a commonly used first-line diagnostic tool in modern cardiological clinical practice.^1^ It provides a low-risk and low-cost examination opportunity to detect thromboses, regional wall motion abnormalities, aorta dissections, pericardial tamponade, valve diseases and other pathological findings.^1^^,^^2^ TTE has a wide clinical application, but it is user-dependent because the physician must be able to perform the examination, consider tentative diagnoses and put findings in the context of the clinical presentation of the patient.^3^ A high level of cognitive and technical skills is needed to perform a reliable TTE, meaning a standardised training program is essential to ensure quality and safety for patients.^1^^,^^3^ Traditionally, competencies in TTE are developed through rotations and fellowship experience consisting of direct observations of colleagues performing TTEs, medical interviews and courses with exams. This approach to longitudinal clinical experience is a less effective way to help medical learners achieve key competencies compared to contemporary educational technologies such as competency-based education.^4^ Because it might be difficult for trainees and departments to prioritise time for education and evaluation, simulation-based training is a beneficial alternative.^5^ Virtual reality (VR) simulation can improve education and transfer skills effectively to clinical performance in other procedures such as laparoscopic cholecystectomy.^6^^, ^^7^ Currently, the evidence on the transfer of VR ultrasound skills to clinical performance is limited. Increasing difficulty, high-risk cases and exposure to rare cases can be performed without compromising the safety and discomfort of patients. Additionally, VR simulation reduces the time that an expert's supervision is needed by providing automatic feedback based on a trainee's score.^5^^, ^^6^^, ^^8^

Mastery learning programs, including a final test, are associated with large effects on knowledge and skills.^4^^,^^9^^,^^10^ The test ensures that every trainee reaches the same level of competency, regardless of their learning pace, by securing clear objectives for trainees assessed by fixed standards and measurements.^4^^,^^8^ A good test is a prerequisite for any mastery learning program where it directs the training and ensures final competencies. However, validity evidence must be gathered before integrating the test into a fixed program.^11^ To our knowledge, no study has previously gathered evidence for a simulation-based test to assess basic competencies in TTE. This study aimed to develop and gather the validity evidence for a simulation-based assessment tool in TTE and establish a credible pass/fail score.

## Methods

### Setting

This study took place at the Simulation Center (SimC) at Odense University Hospital, Region of Southern Denmark, and the Department of Anesthesiology and Intensive Care at Odense University Hospital, Svendborg, Denmark. Data were collected from December 2019 to April 2020. In both departments, the same simulator was installed in a separate room to minimise the risk of disturbances.

### Validity evidence

The principles and framework of Messick were used to gather the validity evidence for the test, including five sources of evidence: content, response process, internal structure, relationship to other variables, and consequences.^11^^-^^13^ Table 1 shows the sources, how they are accommodated and descriptive statistics.

### Simulator and TTE module

The ultrasound simulator resembles an ultrasound machine with a mannequin torso, a touch screen and a sector probe. A dynamic VR simulation image is shown on the screen when the torso is scanned with the probe. The simulator allows trainees to practise and develop ultrasounds skills by presenting clinical cases and evaluating the student with feedback on sonographic skills and pathological findings. The software of the ultrasound simulator was not updated during the data collection to ensure the same conditions for all participants.

### Test content

An expert in cardiology (JHC) and two simulation experts (MSN and ABN) evaluated which knowledge and skills, together with anatomical structures and pathological patterns, are essential to perform a reliable TTE. Based on the experts' opinions, the clinical relevance of the simulator's diagnostic cases was assessed for clinical applicability, securing the test content. All available cases were assessed before consensus was reached on a full test, including an introductory case with a healthy patient (case 1) and two diagnostic cases with patients with acute myocardial infarction (case 5) and mitral insufficiency (case 9). Finally, the participants had to identify correct anatomical structures in three different projections.

**Table 1:** Source of evidence framework according to Messick

Source	Description	Plan	Analysis
Content	Ensure that the test content reflects what it is intended to measure	Expert determination of content in conjunction with international guidelines	
Response process	Ensure uniformity and control of the response process and minimalise assessment bias	Standardise written information and answer sheet, same instructor for all completions	
Internal structure	Relationship among data items within the instrument and underlying construct	Calculate internal consistency reliability	Cronbach's a and intraclass correlation coefficient (ICC)
Relationship to other variables	Extent to which assessment results relate to other variables	Compare the scores between the groups (novices, intermediates, experienced)	ANOVA with Bonferroni correction
Consequences	Evidence pertaining to intended and unintended consequences of passing and failing	Establish a pass/fail score and explore consequences of this score in terms of false-positives and false-negatives	Contrasting-groups method

### Participants

Physicians were invited to participate in the study either by email or verbally and received written and verbal information regarding the study. Acceptance of the use of data was a term for participation.

We aimed to include a minimum of 10 participants in each group to meet the assumption of normally distributed data in medical education research.^14^

Participants were divided into three groups based on their experience with TTE. All participants were physicians from hospitals in the Region of Southern Denmark. The novice group included physicians with a maximum of 19 self-performed TTEs. The intermediate group was physicians who had performed 20–200 TTEs, and the experienced group was physicians who had performed more than 1000 TTEs. An anonymous study ID was given to each participant. The participants received no compensation or salary.

An application for ethical approval was sent to the regional Scientific Ethics Committee in the Region of Southern Denmark, where it was concluded that no further applications were needed. All data were entered and handled in an online database: the Research Electronic Data Capture (REDCap), hosted by the Open Patient Data Explorative Network (OPEN). Only MSN had access to the data, and all interactions in the database were logged.

**Table 2:** Mean Scores, Standard Deviations, and Confidence Intervals of each Case

Group	n	M	SD	95% CI
Case 5 Projections				
Novices	16	7.9	3.4	[6.1, 9.7]
Intermediates	10	13.6	4.7	[10.2, 17]
Experienced	10	16.8	0.4	[16.5, 17.1]
Case 9 Projections				
Novices	16	7.2	2.9	[5.6, 8.7]
Intermediates	10	13.1	5.0	[9.5, 16.7]
Experienced	10	17.0	0.0	[17.0, 17.0]
Case 5 Clinical Conclusion				
Novices	16	1.3	0.7	[0.9, 1.6]
Intermediates	10	1.5	0.7	[1.0, 2.0]
Experienced	10	2.0	0.0	[2.0, 2.0]
Case 9 Clinical Conclusion				
Novices	16	0.6	0.6	[0.2, 0.9]
Intermediates	10	1.3	0.7	[0.8, 1.8]
Experienced	10	2.0	0.0	[2.0, 2.0]
Anatomy Quiz: Score				
Novices	16	8.4	1.9	[7.4, 9.4]
Intermediates	10	10.0	2.9	[7.9, 12.1]
Experienced	10	11.9	0.3	[11.7, 12.1]

### Completion of the test and data collection

Validity evidence on the response process was ensured by standardising the test for all participants. Each participant was informed of the aim of the study and how the data were used, followed by an introduction to the simulator by MSN. The data collection was conducted in one session for each participant, consisting of two simulation-based cases and one anatomical test.

Following the introduction, the participant began the first case, which was not part of the test program. Case 1 did not present any pathological findings and thus showed normal sonographic findings. This was to ensure the participant felt confident using Doppler mode and gain and contrast adjustments and knew how to freeze the image when the requested projection was performed.

The test program started with a virtual patient case with a stationary left ventricle and regional wall-motion abnormality, implicating an acute myocardial infarction (case 5). Participants were requested to identify the following 17 projections: the parasternal long axis, the parasternal long axis with Doppler on the mitral valve, the parasternal long axis with Doppler on the aortic valve, the parasternal short axis with papillary muscle, the parasternal short axis with the aortic valve, the parasternal short axis with Doppler on the aortic valve, apical 4  chambers, apical 4 chambers with Doppler on the mitral valve, apical 2 chambers, apical 3 chambers, apical 3 chambers with Doppler on the mitral valve, apical 5 chambers, apical 5 chambers with Doppler on the mitral valve, apical 5 chambers with Doppler on the aortic valve, apical 5 chambers with a continuous wave, subcostal 4 chambers, and subcostal inferior vena cava. During the test, participants froze the screen when they found the optimal place for the requested projection. The participant was then asked to estimate an ejection fraction (EF). Finally, the participant had to suggest a pathological diagnosis. The request for each target projection was read aloud by MSN, following the same structure for every participant. Participants verbally stated when they found the requested projection. The second case was a 9-year-old boy where sonographic findings revealed a leak over the mitral valve, suggesting a mitral insufficiency (case 9). After the final projection in each case was performed, answers were locked, and participants were not allowed to scan further. In the last part of the test, participants were exposed to an anatomical quiz. No evaluation occurred while the test was performed.

### Statistical analysis

The projections were continuously evaluated by JHC and MSN, attaining a score of either correct or incorrect. The scores were noted by MSN. The cumulative maximum score of the test was 50 points, with 1 available point for each correct projection, EF, diagnosis and anatomical structure.

The test scores were used to explore whether the test could distinguish between novice, intermediate and experienced physicians. The group's mean scores were compared using a one-way analysis of variance with Bonferroni correction for multiplicity. Cronbach's alpha was calculated as a measure of internal consistency reliability and the intraclass correlation coefficient (ICC) to assess performer consistency. We established a pass/fail score based on the contrasting groups' standard method, and the consequences in terms of false positives and false negatives were explored. Statistical analyses were performed using SPSS. All statistics were considered at a significance level of 5%.

## Results

Thirty-six participants were included in the study: 16 novices, consisting of 14 anaesthesiologists (88%), one physician with a speciality in acute medicine (6%) and one cardiologist (6%); 10 intermediates, including six anaesthesiologists (60%) and four cardiologists (40%); 10 experienced physicians, including nine cardiologists (90%) and one anaesthesiologist (10%).

### Internal structure

The internal consistency reliability of case 5 showed an Alpha = .93; 95%CI [.89, .96]. The same internal consistency reliability was reached for case 9 (Alpha = .93; 95%CI [.90, .96]). An even higher Cronbach's alpha was retrieved when the results from projections in each case were compared (Alpha = .97; 95% CI [.95, .99]).

The ICC for every projection in a single case was r_(__35)_ = .95, p <.001. The ICC on all parameters for both cases was r_(__35)_ = .81; 95%CI [.69, .89],  p <.001, which shows a relatively high consistency of the performer. The ICC for every projection in both cases calculated together was r_(__35)_ = .97; 95% CI [.95, .99], p <.001 which is an expression of how consistent the participant is. Therefore, the risk of a performer achieving a high score through luck is very low. The lowest internal consistency reliability was seen in the anatomy quiz (Alpha = .81; 95%CI [.72, .90]). For the complete test content, including projections, estimated EF, diagnoses for both cases and score for the anatomical quiz, alpha = .88; 95%CI [.80, .94].

### Relationship to other variables

The mean scores of each case are presented in Table 2. The mean score for novices was 7.9 points (SD= 3.4) for projections in case 5, 1.3 points (SD= 0.7) for case 5 conclusions, 7.2 points (SD= 2.4) for case 9 projections, 0.6 points (SD= 0.6) for case 9 conclusions, and 8.4 points (SD= 1.9) for the test in anatomical structures.

The group of intermediate physicians scored a mean of 13.6 points (SD= 4.7) for projections in case 5, 1.5 points (SD= 0.7) for conclusions on case 5, 13.1 points (SD= 5.0) for case 9 projections, 1.3 points (SD= 0.7) for case 9 conclusions and 10.00 points (SD= 2.9) in the anatomical structures.

The mean score for experienced physicians was 16.8 points (SD= 0.4) in case 5 projections, 2.0 points (SD= 0.0) for case 5 conclusion, 17.0 points (SD= 0.0) for case 9 projections, 2.00 points (SD= 0.0) for case 9 conclusions, and 11.9 points (SD= 0.3) for anatomical structures.

The Bonferroni post hoc test proved a difference between novice and experienced physicians on all parameters (Table 3). A significant difference between the novice and intermediate groups was observed on the parameters, except for the case 5 conclusion and the test in anatomical structures (Table 3).

### Consequences

Using the standard-setting method of the contrasting groups, a pass/fail standard score of 48, 95% CI [46.6, 48.6] was established based on the mean test score of novice and experienced physicians. As a result, all experienced physicians and two intermediate physicians managed to pass the test. However, none of the novices passed. No false negatives or false positives occurred.

## Discussion

This study provided evidence of the validity of a simulation-based test as an assessment tool to ensure basic competency in TTE. Using only one case, this can be assessed reliably and validly to conclude participants' skill levels. The test could differentiate between novice and experienced physicians on all parameters. To our knowledge, no studies have gathered the validity evidence for a simulation-based test to ensure basic competencies in TTE.

**Table 3:** Bonferroni multiple comparisons test indicating significant differences in performance between the groups

Group (I)	Group (J)	M_I _-M_J_	p	95 % CI
Case 5 Projections				
Novices	Intermediates	-5.7	<.001	[-9.1, -2.2]
	Experienced	-8.9	<.001	[-12.3, -5.5]
Intermediates	Experienced	-3.2	.124	[-7.0, 0.6]
Case 9 Projections				
Novices	Intermediates	-5.9	<.001	[-9.3, 2.6]
	Experienced	-9.8	<.001	[-13.2, -6.5]
Intermediates	Experienced	-3.9	.037	[-7.6, -0.2]
Case 5 Clinical Conclusion				
Novices	Intermediates	-0.3	.903	[-0.9, 0.4]
	Experienced	-0.8	.010	[-1.4, -0.2]
Intermediates	Experienced	-0.5	.201	[-1.2, 0.2]
Case 9 Clinical Conclusion				
Novices	Intermediates	-0.7	.007	[-1.3, -0.2]
	Experienced	-1.4	<.001	[-2.0, -0.9]
Intermediates	Experienced	-0.7	.023	[-1.3, -0.1]
Anatomy Quiz: Score				
Novices	Intermediates	-1.6	.175	[-3.6, 0.5]
	Experienced	-3.5	<.001	[-5.5, -1.5]
Intermediates	Experienced	-1.9	.117	[-4.1, 0.3]

Note. MI = mean of group I. MJ = mean of group J

Note. MI - MJ = Difference of means between group I and J

As described by Messick, validity refers to the value and worth of an assessment tool or task, and validation refers to the gathering of data and the analysis of evidence to assess validity.^11^ As shown in Table 1, Messick presented five sources of evidence.^11^

To accommodate validity concerning the content, the development of curriculum and cases were provided under management by an expert in TTE, who also had years of experience teaching TTE. The content contained common ultrasound findings in patients with heart diseases. The chosen setup and curriculum were believed to be representative of the content in question. A limitation of this study was the relatively few experts on the panel. A possible solution to increase the content validity would be using a Delphi-method survey with more panel experts. This method has been used in similar studies and creates a wide agreement between experts regarding content.^15^

To ensure validity evidence for the response process, all participants were introduced to the project and simulator from the same guideline. This created an environment and a setting where standardisation was in focus. The instructor observed the participants during the test, making sure no data went missing. However, they were not allowed to interact during the test, to prevent and minimise potential bias between the instructor and the participant, which could affect the data.

According to Downing and Yudkowsky, the internal consistency of our test is high (Alpha = .88; 95% CI [.80, .94]).^12^ A reliability of alpha = ≥.80 is expected for moderate-stakes assessment.^16^ However, most educational measurement professionals suggest a reliability coefficient of at least alpha = .90 for high-stakes assessments, such as certification examinations in medicine.^16^ Only comparing the projections showed Alpha = .97,  95% CI [.95, .99]. This indicates that the test included a high amount of strength and reliability. This test was intended as an approach for mastery learning, which allows the trainee to repeat training until they consider themselves at an adequate level of competency.

A significant difference existed between novice and experienced physicians (Table 3). As predicted, the mean score increased in relation to the level of experience, but an increase in consistency, as well as a decrease in variance, was observed. A limitation in this context is that no clear definition of experience in validation studies was found in the literature. This could have led to selection bias because the participant's estimate of the number of performed TTEs might be inaccurate. Additionally, the competence quality is not guaranteed to correlate with the number of performed TTEs.

Overall, the study showed that the experienced group constantly performed well and with minimal variation between participants in the group. This was expected because it correlates with the three-step model for acquiring motor skills, as presented by Fitts and Posner.^17^ Fitts and Posner presented three sequential stages of learning, where movement eventually becomes automatic as competency is gathered. In the first stage, the cognitive stage, individuals use their working memory and declarative knowledge. This was confirmed by observing the participants in the novice group. In general, they used more time and often struggled with finding the correct projections. The second stage is called the associative stage. It is characterised by a decrease in the dependence on working memory and results in a more fluent movement. The last stage is autonomous and requires minimal cognitive effort as the movement becomes an automatised routine, which creates a greater ability to detect errors, along with better decision-making and improved anticipation, the sum of which is minimal variations and errors.^18^^-^^20^ Our observations of the experienced physicians and their scores and test times showed that they had all reached the last learning phase. The time a trainee spends in each stage depends on their level of skills, knowledge and behaviours. Supporting each learning pace with no time restraint is essential in the educational setup because it allows trainees with different learning paces to reach the same skill levels.^17^

Ultrasound is a clinical tool that, in the last decade, has proven increasingly useful in a wide range of specialities. Improved performance in diagnostic ultrasound scanning is found when learning by simulation-based mastery training.^21^ Studies show the efficacy of mastery learning programs when gaining skills in ultrasound, as well as the ability to differentiate between competency levels of ultrasound examiners.^20^^, ^^22^^, ^^23^ Multiple ultrasound simulation-based tests with established evidence of validity are included in the certification of physicians in a broad range of specialities. An example is the European Respiratory Society, which requires all trainees to pass a simulation-based test before moving to the next step in their standardised training and certification program for an endobronchial ultrasound.^24^^-^^27^ This approach is recommended in the international guidelines.^28^

### In cardiology

Studies suggest that competencies in the simulation of cardiology procedures can translate to the operator's skills in clinical practice because more experienced clinicians perform better in a simulation.^29^^,^^30^ The role of TTE simulation in training clinicians has proven useful in a few studies, but to the best of our knowledge, no assessment tool has been developed yet. Simulation-based TTE training has proven more efficient than traditional didactic methods (lectures and videos) for teaching basic TTE skills to anaesthesiology residents.^31^ TTE simulation has also proven useful in the training of sonographers when participants develop image acquisition skills.^32^ This study differs from other studies because the focus is on developing an assessment tool as well as gathering competencies in TTE. We focused on reaching a specific level, using mastery learning, and not proving the usefulness of simulation-based training because the evidence is already clear regarding this. The same approach to developing competencies is likewise used in other ultrasound procedures.^20^^-^^22^

TEE is another diagnostic procedure in the cardiological speciality where operator skills are essential. TEE is well-studied in terms of simulation-based training compared to TTE. Simulation-based learning in TEE has proved significantly better compared to e-learning and hands-on training, and novice operators acquire TEE views faster and with better quality after TEE simulator-based training, in comparison to lecture-based training.^33^^-^^36^ These studies are limited to showing that simulation training improves skills in a simulation setting.^33^^-^^36^ However, other studies have managed to show that simulation-based TEE training can improve competencies in a clinical setting.^24^^,^^37^ In comparison to TTE, TEE has a validated simulation-based test for assessing key competencies.^38^ This raises considerations regarding the possibility of implementing TTE simulation-based tests and training equally to TEE.

Simulation-based training and assessment provide the possibility of training without risk, discomfort or unnecessary time consumption for patients. By gathering competencies in TTE, we provided the opportunity to gain a basic skill level before approaching the clinic. More studies are desired to determine the performance and learning curves of novices with TTE. Even though we managed to include more than 10 participants in each group, the generalisability would improve if the study groups were larger and included international participants. A study in a clinical setting with a focus on competence development is also needed. This could include an assessment of diagnostic decision-making and how to handle the ultrasound device, together with further diagnostics and treatment of clinical findings. This test focused on scanning and identifying pathological findings. Other factors are important to gain an optimal examination of the patient, such as patient communication. A limitation of this test is that it does not consider differential diagnostic skills or related clinical knowledge. Importantly, initial clinical supervision is still needed after completing a simulation-based mastery program.

## Conclusions

This newly developed VR simulation-based test for assessing skills in TTE showed good reliability and could discriminate between participants with different levels of TTE experience. The established pass/fail standard resulted in zero false negatives or false positives. This standardised test could act as an important prerequisite in a mastery learning training program and as a supplement to clinical learning, securing higher quality and improved skills for physicians before clinical decisions are made based on TTE. This study also leads the way for further studies determining the performance and learning curves of novices in TTE.

### Conflict of Interest

The authors declare that they have no conflict of interest.
